# Experience With Direct-to-Patient Recruitment for Enrollment Into a Clinical Trial in a Rare Disease: A Web-Based Study

**DOI:** 10.2196/jmir.6798

**Published:** 2017-02-28

**Authors:** Jeffrey Krischer, Peter F Cronholm, Cristina Burroughs, Carol A McAlear, Renee Borchin, Ebony Easley, Trocon Davis, Joyce Kullman, Simon Carette, Nader Khalidi, Curry Koening, Carol A Langford, Paul Monach, Larry Moreland, Christian Pagnoux, Ulrich Specks, Antoine G Sreih, Steven Ytterberg, Peter A Merkel, Vasculitis Clinical Research Consortium

**Affiliations:** ^1^ Rare Diseases Clinical Research Network Data Coordinating Center Health Informatics Institute University of South Florida Tampa, FL United States; ^2^ University of Pennsylvania Philadelphia, PA United States; ^3^ Vasculitis Foundation Kansas City, MO United States; ^4^ Mount Sinai Hospital Toronto, ON Canada; ^5^ St. Joseph's Healthcare Hamilton Hamilton, ON Canada; ^6^ University of Utah Salt Lake City, UT United States; ^7^ Cleveland Clinic Foundation Cleveland, OH United States; ^8^ Boston University School of Medicine Boston, MA United States; ^9^ University of Pittsburgh Pittsburgh, PA United States; ^10^ Mayo Clinic Rochester, MN United States; ^11^ Vasculitis Clinical Research Consortium Philadelphia, PA United States

**Keywords:** clinical trial, research subject recruitment, social media, direct-to-consumer advertising, granulomatosis with polyangiitis

## Abstract

**Background:**

The target sample size for clinical trials often necessitates a multicenter (center of excellence, CoE) approach with associated added complexity, cost, and regulatory requirements. Alternative recruitment strategies need to be tested against this standard model.

**Objectives:**

The aim of our study was to test whether a Web-based direct recruitment approach (patient-centric, PC) using social marketing strategies provides a viable option to the CoE recruitment method.

**Methods:**

PC recruitment and Web-based informed consent was compared with CoE recruitment for a randomized controlled trial (RCT) of continuing versus stopping low-dose prednisone for maintenance of remission of patients with granulomatosis with polyangiitis (GPA).

**Results:**

The PC approach was not as successful as the CoE approach. Enrollment of those confirmed eligible by their physician was 10 of 13 (77%) and 49 of 51 (96%) in the PC and CoE arms, respectively (*P*=.05). The two approaches were not significantly different in terms of eligibility with 34% of potential participants in the CoE found to be ineligible as compared with 22% in the PC arm (*P*=.11) nor in provider acceptance, 22% versus 26% (*P*=.78). There was no difference in the understanding of the trial as reflected in the knowledge surveys of individuals in the PC and CoE arms.

**Conclusions:**

PC recruitment was substantially less successful than that achieved by the CoE approach. However, the PC approach was good at confirming eligibility and was as acceptable to providers and as understandable to patients as the CoE approach. The PC approach should be evaluated in other clinical settings to get a better sense of its potential.

## Introduction

Despite the emphasis given to randomized controlled trials (RCTs) as the gold standard for the evaluation of new and promising therapies, it is well recognized that sufficient numbers of potential study participants are usually not available at a single institution, necessitating the organization of multicenter studies, development of specialized infrastructure (eg, study staff, training of site personnel, data transfer practices), layers of additional administrative work (eg, multi-institutional agreements and subcontracts, applications to multiple institutional review boards [IRBs], investigator meetings), and all the effort required to maintain study team cohesiveness and momentum across multiple sites. Yet, even with such high levels of investment, accrual into RCTs often fails to meet enrollment goals, even after enrollment periods are markedly extended. As many as 40% of all trials and 71% of phase 3 trials supported by the National Cancer Institute’s clinical trials program fail to ever achieve their target accrual [1,2]. Concern over the failure to achieve targeted accrual numbers extends to all trials nationally, with 19% of RCTs closing without having achieved at least 85% of their target accrual [3]. Among reasons significantly associated with unsuccessful accrual are increased number of eligibility requirements (presumably limiting the number of eligible individuals), less number of research sites, nonindustry funding, and a nonplacebo comparison arm.

Expanding the number of treatment sites in a traditional center of excellence (CoE) model for conducting RCTs is associated with significant costs. Even if multiple sites can be coordinated, a major problem with the traditional CoE model is that the majority of potential study participants are still out of reach of the few geographically-limited clinical centers involved in the trial. This is especially the case in trials targeting individuals with rare diseases [4]. Catchment areas and referral patterns are often related to a lack of experts in the disease under study, institutional competition, insurance agreements, or personal referral networking among physicians. Novel ideas to reach a larger and perhaps less preselected population could reduce the time and cost of clinical trials, increase the generalizability and social value of their findings, and increase the likelihood that important clinical questions could be addressed more quickly and successfully to better support advancing new beneficial therapies to affected individuals. Even more importantly, such methods would revolutionize clinical research in rare diseases.

These factors motivate the consideration of novel methods to recruit potentially eligible individuals to RCTs. The pharmaceutical industry makes extensive use of direct-to-consumer advertising of approved prescription drugs [5,6] with a presumed objective to attract patients to their drugs, as well as to inform about treatment alternatives and encourage communication with health care providers. Although regulated by the Food and Drug Administration (FDA), this marketing strategy is not without its detractors, especially when marketing begins soon after approval and there remains a need to educate providers on the appropriate use of the drug, and more data on possible untoward side effects is needed [7].

Although investigators rarely have the budget to employ the same advertising strategies as does the pharmaceutical industry, the rapid growth of the Internet and social media provide inexpensive, and in some cases, free access to millions of potential research participants. The value of advertising over the Internet is a major business strategy and investigators have taken note to use this methodology to broaden their reach to potentially eligible individuals and simultaneously overcome the barriers to enrollment into RCT [8-14].

This paper describes an effort to test whether direct recruitment of study individuals using social marketing strategies (ie, Web-based tools, online patient communities, Facebook, Twitter, Google+, others) to reach a target population and provide an interactive Web-based method to engage, educate, enroll, and obtain informed consent, provides a viable option for recruitment and enrollment into an RCT. Notably, the approach also included elements designed to reduce known barriers to enrollment [15] by having only one IRB, reducing study burden by not requiring treating physicians to be involved in the research, and asking an important clinical question, with 2 equally attractive treatment arms that were considered accepted standards of care.

## Methods

### Rationale for The Assessment of Prednisone in Remission (TAPIR) Trial

To test direct patient recruitment (patient-centric, PC) and compare it with the traditional CoE recruitment model, a clinical trial was designed for use as the study setting. The Assessment of Prednisone in Remission (TAPIR) trial tests whether patients with granulomatosis with polyangiitis (GPA; Wegener’s) have better outcomes after their GPA is well-controlled (in remission) if they stay on a maintenance dose of 5 mg/day of prednisone or fully come off of prednisone (0 mg/day; Clinicaltrials.gov NCT01940094 and NCT01933724).

Studies in the last 20 years have addressed the use of immunosuppressive medications in GPA. Unlike immunosuppressive medications, the use of prednisone has not been rigorously evaluated. There is little evidence to guide the use of prednisone and there is considerable practice pattern variation, especially after the induction of remission. Of particular debate is whether low-dose prednisone contributes to maintaining the remission of GPA. Some experts support the use of long-term, low-dose prednisone, claiming improved disease control, a subsequent reduction in the exposure to toxic immunosuppressive medications, fewer periods of exposure to high-dose prednisone, and a reduction in the accumulation of disease-related scarring. Others argue that the use of long-term, low-dose prednisone is ineffective at reducing relapses and exposes patients to the potential toxicity of high cumulative doses of prednisone. The efficacy of long-term, low-dose prednisone for the treatment of GPA to prevent relapses or reduce treatment-related toxicity is a matter of continued debate [16].

### Trial Setting

The setting for this RCT is the Vasculitis Clinical Research Consortium (VCRC) **,** a founding member of the Rare Diseases Clinical Research Network (RDCRN) [17] and the major clinical research infrastructure in North America for the study of vasculitis. The work conducted by the VCRC includes clinical trials, outcome measures development, large cohort and clinical epidemiologic studies, biospecimen collection and repository, translational investigations including biomarker discovery and genomics, and research training. The US and Canada VCRC vasculitis clinical centers (ie, CoE sites) participated in this study. Simultaneously, the PC approach has been implemented by the VCRC and the RDCRN Data Management and Coordinating Center (DMCC) for cross-sectional studies and patient communication using Web-based tools and the Vasculitis Patient Contact Registry, which consists of nearly 3000 individuals in the United States with vasculitis, of whom 1458 report a diagnosis of GPA.

### The TAPIR Protocol

To be eligible, patients must have an established diagnosis of GPA (verified by medical record review by protocol oversight management team) and meet at least two of the 5 modified American College of Rheumatology (ACR) criteria [18]. Patients must have had active disease within the prior 12 months (initial presentation or relapse) that at time of active disease required treatment with prednisone ≥ 20 mg/day. At the time of enrollment, the patient’s disease must be in remission and their prednisone dose ≥ 5 mg/day and ≤ 20 mg/day. If the patient is taking an immunosuppressive medication agent other than prednisone (maintenance agent) then the maintenance agent must be at a stable dose for 1 month prior to enrollment with no plans by the treating physician to change the dose (other than for safety purposes or toxicity) for the duration of the study (through the month 6 visit or early termination). Participants must be age 18 years or above and their treating physician needs to agree that either treatment assignment is standard of care.

**Figure 1:**
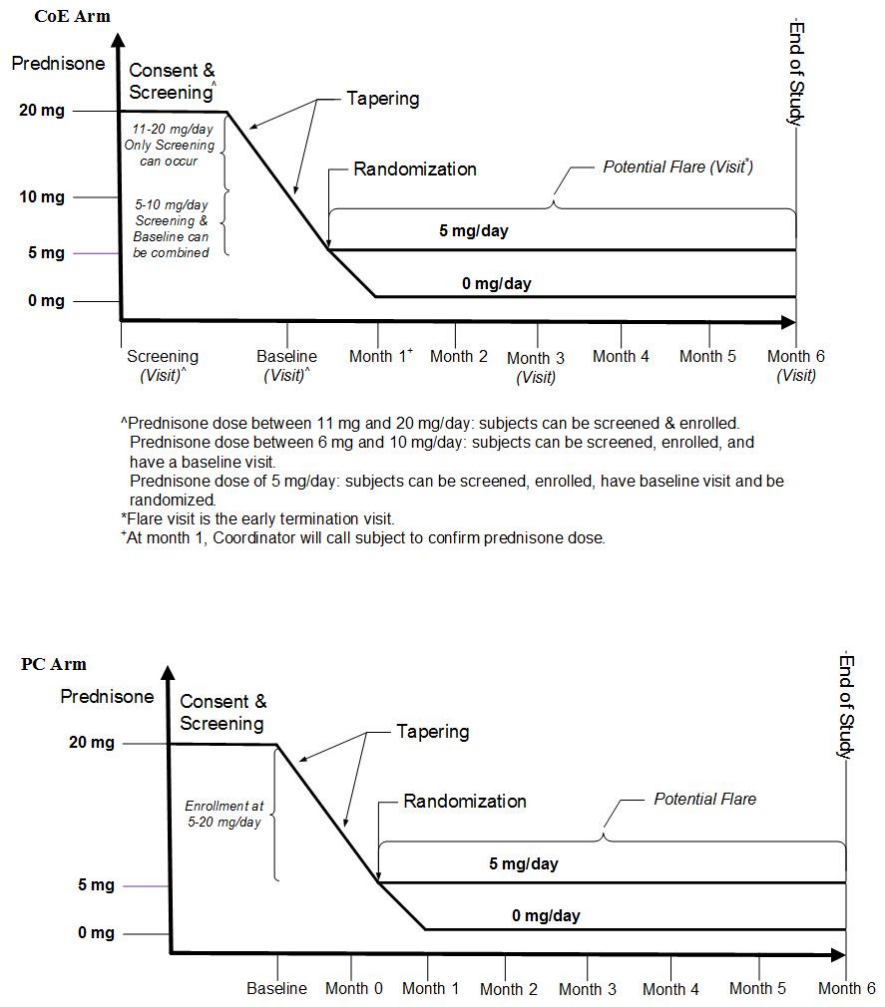
The Assessment of Prednisone in Remission (TAPIR) study summary for center of excellence (CoE) and patient-centric (PC) arms.

All enrolled participants are tapered from their baseline prednisone dose to 5 mg/day and are then randomized using a 1:1 ratio to remain on 5 mg/day or taper their prednisone dose down to 0 mg/day for the duration of the study (approximately 6 months) or until a study endpoint (Figure 1). All study participants are followed for 6 months or until an increase of prednisone dose (after randomization) occurs, whichever comes first. CoE participants have up to 4 study visits, a screening visit (visit 1), a baseline (visit 2), a month 3 visit (visit 3), and a month 6 or flare visit (visit 4), and up to 2 follow-up phone calls from the study coordinator at randomization and at month 1 (randomization and month 1 phone call may be combined if randomization occurs at month 1).

The primary study endpoint outcome at 6 months included: either (1) continued remission; or (2) a relapse, defined as the physician decision to increase glucocorticoids for disease relapse. The secondary study endpoints include: rates of flare subtypes (severe vs nonsevere), time to event (flare), health-related quality of life, safety (adverse events), and protocol performance (compliance, retention, data completeness, timeliness of data entry, and data accuracy).

### Patient-Centric Recruitment

**Figure 2:**
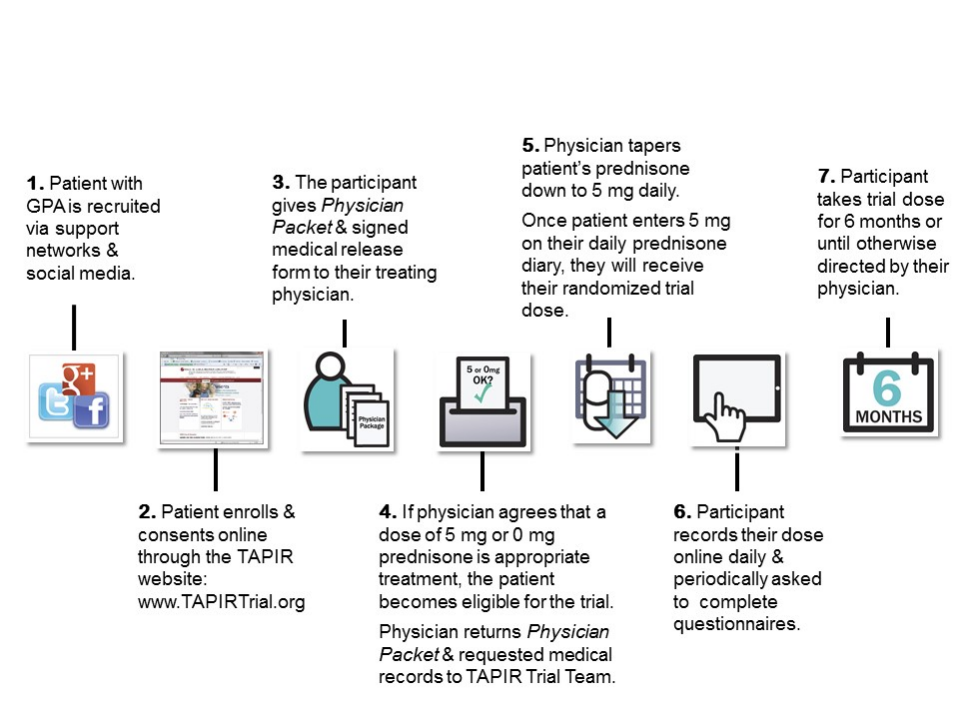
Patient-centric (PC) arm study flow.

The VCRC Contact Registry, social media websites, and the Vasculitis Foundation, the largest patient advocacy group for vasculitis, are utilized to direct patients to the study public website (Figure 2). The study’s public website provides information including inclusion or exclusion criteria, requirements for participation in the study, the study design of tapering of prednisone to 0 mg/day or 5 mg/day, and whom to contact with questions.

The public TAPIR website also contains an interactive informed consent form to enroll in the study. Potential participants are presented with a video about the study that explains the goals of the research and the risks and benefits of the study. Participants are able to access this video continually during the consenting process and after enrollment. The participant is able to contact study staff through social media or other options including phone or email. Once enrolled in the study, the participant has access to a study website that is personalized for each participant. Participants can keep track of their progress in the study, access the Web-based consent form, and access study forms. Participants receive their treatment randomization assignment via the participant website as well as a physician packet that the participant takes to his or her treating physician explaining the research study and the participant’s involvement in the study. The physician has the option to agree to be contacted by study staff to answer questions about their role in this research study.

In order for the participant to be eligible for the study and to establish that the treating physician is not engaged as a researcher in the study, as defined by the US Office for Human Research Protections, the treating physician needs to agree that either a prednisone dose of 5 mg/day or 0 mg/day is standard of care; such agreement is thus consistent with the treating physician providing routine care. A protocol oversight management team provides independent review of a patient’s medical records provided by the treating physician to determine if the participant meets eligibility requirements. Participants taper their prednisone dose under the guidance of their own treating physician. Once the participant reaches a dose of prednisone of 5 mg/day, the participant reengages the website and is randomized to continue prednisone at 5 mg/day or taper prednisone to 0 mg/day. The participant’s treating physician is then notified of the participant’s randomized dose. Participants are to be followed for 6 months from randomization.

### Center of Excellence Recruitment Model

Investigators at CoE sites are responsible for implementation of the protocol, including screening of potential participants, enrollment of participants, conduct of the protocol, and adherence to federal and local guidelines for clinical research and the protection of human participants (IRB approval). Site-specific CoE study coordinators are responsible for managing the day-to-day operations and implementation of the TAPIR protocol, including the completion of all relevant documentation and record-keeping; scheduling participants for study visits and maintaining the visit calendar; collecting the prednisone study diary; following principles of Good Clinical Practice; submitting the protocols and consent forms to the institutional review or ethics boards; and assisting with patient education and training.

Recruitment occurs through the clinical practices of each site. Participants are also recruited, as needed, via mailings to appropriate clinicians in the investigators’ catchment area. Details of the goals of the research and the risk and benefits of the protocol are reviewed with each potential study participant. The consenting process is documented in the study chart.

An optional part of the study was participation in a 21-question knowledge assessment to compare the Web-based PC-arm based informed consent with the standard administration of an informed consent in the CoE. The knowledge assessment contained questions regarding the purpose of the study, eligibility, treatment arm dosing, risks, and communication with the study team or the individual’s treating physician.

### Qualitative Interviews

Semistructured qualitative interviews were conducted with patients with GPA in both the CoE and PC arms. Patients were interviewed following the consent process and at any of 3 study endpoints (6-month completion, flare related drop-off, nonflare related drop-off). Regardless of study arm, patient interview domains included: (1) factors affecting the decision to enroll, (2) motivation for participation, (3) perception of the recruitment or consent process, (4) expectations for participation, including risks and benefits, and (5) understanding and comfort with tapering period or process. Additional questions were added for participants in both study arms that did not reach the 6-month completion endpoint.

Participant interviews were conducted over the phone. With the participant’s permission, all interviews were audio recorded and professionally transcribed. All deidentified qualitative data was entered into NVivo 10 (QSR International), qualitative data analysis software, to facilitate analysis. Interviews were analyzed using modified grounded theory techniques. After a close reading of the initial transcripts, a codebook was developed comprised a priori and grounded theory codes. This analytical method allowed the team to code for ideas of particular interest to the study team and to code for ideas that emerged from the participants’ words. Twenty percent of all transcripts were coded by 2 coders in order to assess interrater reliability. If there was less than 90% agreement or .6 kappa in coding, the coders and investigators discussed the areas under question and discrepancies were resolved by consensus methods.

## Results

### Enrollment Findings

The PC and CoE recruitment efforts were launched on February 17, 2014, after receiving IRB approval. The CoE effort was initiated at the 2 sites that had received IRB approval at this point in time. There was a 2-month lapse between the activation of the first 2 CoE sites and the eighth and final CoE site. Distribution of recruiting materials by the Vasculitis Foundation also began on February 17, 2014; this included promotion of the study on their website, creation of a separate TAPIR trial Web page, inclusion in their quarterly newsletter, a webinar on February 15, 2015, and mention at Chapter meetings and the 2015 Vasculitis Foundation symposium. Distribution to the 1458 patients with GPA in the United States enrolled in the Vasculitis Patient Contact Registry began on May 13, 2014, and was repeated in one-to-two month intervals.

**Figure 3:**
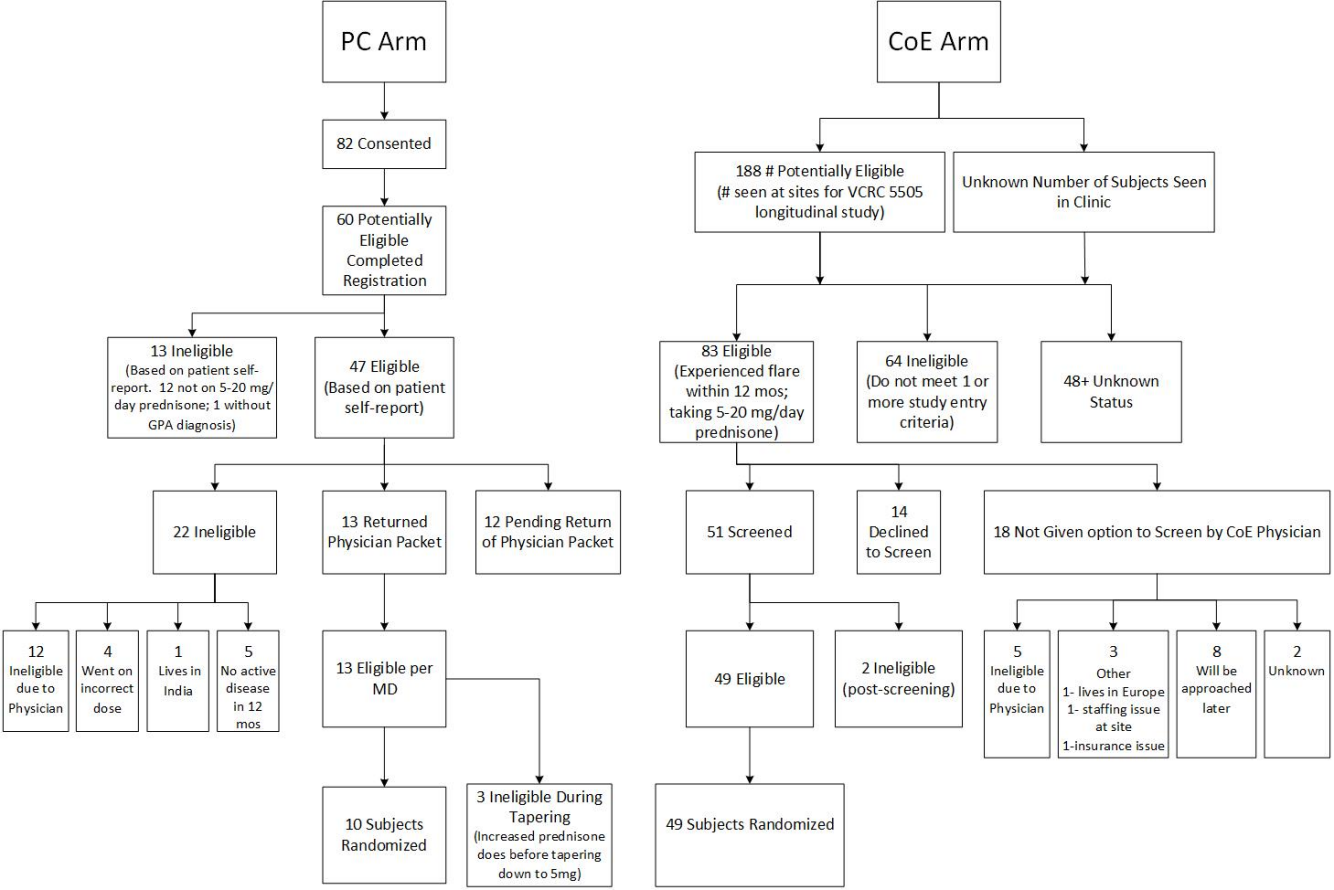
The Assessment of Prednisone in Remission (TAPIR) trial consort diagram.

Enrollment, as of May 31, 2016, was 49 in the CoE and 10 in the PC arms. Planned enrollment was 3.3 participants per month for each of the arms, whereas the actual enrollment rate was 0.4 and 1.8 participants per month for the PC and CoE arms, respectively. The social media–directed recruitment effort brought 16,094 individuals to the TAPIR trial website over this time period. Of this large group, only 82 (0.5%, 82/16,094) consented to participate in the study (Figure 3). Information on diagnosis and prednisone dosing was provided during registration by 60 of these 82 (73%; 60/82). Self-reported data suggested 47 of the 60 (78%; 47/60) were potentially eligible for enrollment. The distribution of demographic and GPA diagnosis clinical data for participants enrolled is summarized in Table 1.

**Table 1:** Study participant demographics and medical history.

Demographics		Patient-centric arm (n=10)	Center of excellence arm (n=49)
**Age at enrollment (years)**			
	Mean	54.8	55.6
	Median	56.5	59
	Range	37-69	21-80
			
**Sex**			
	Male	3	25
	Female	7	24
	Not indicated	0	0
**Age at diagnosis (years)**			
	Mean	47.7	53.1
	Median	47	56
	Range	31-65	20-80
			
**Newly diagnosed versus recurrent disease**			
	Newly diagnosed	1	28
	Recurrent disease	9	21
**Years since diagnosis**			
	Mean	8.4	3.4
	Median	7	2
	Range	1-24	0-23

After review of medical records, 64 of the 188 (34%; 64/188) potential CoE participants were found to be ineligible as compared with 13 of 60 (22%; 13/60) in the PC arm (*P*=.10). The 2 approaches toward recruitment were not significantly different in provider acceptance with 18 of the 83 (22%; 18/83) eligible participants on the CoE arm excluded by their treating physician as compared with 12 of 47 (26%; 12/47) on the PC arm (*P*=.78). The actual enrollment of those confirmed eligible by their physician was 10 of 13 (77%; 10/13) and 49 of 51 (96%; 49/51) in the PC and CoE arms (*P*=.11), respectively.

In the PC arm, those who were not eligible were not on the appropriate prednisone dose (n=12) or did not have GPA (n=1). Those who were eligible were requested to contact their treating physician to confirm eligibility and obtain physician concurrence that the treatment plan of either 5 mg/day or 0 mg/day of prednisone was acceptable (n=47). Despite a multipronged approach or reminders and attempted direct contact with treating physicians by study staff, this step posed a significant obstacle with 26% of consenting eligible participants unable to proceed with the study due to the inability to get physician concurrence. Of the 35 physician packets returned, an additional 22 people (63%, 22/35) were found to be ineligible for various reasons (Figure 3), leaving 13 eligible participants. Of those, 10 were randomized to one of the 2 TAPIR treatment arms. By comparison, the CoE sites identified 147 potentially eligible individuals of whom 83 (57%, 83/147) met the TAPIR trial eligibility criteria. 51 of 83 (61%; 51/83) were found to be eligible; 49 of these 51 patients were randomized.

At present, none of the 10 participants (0%) on the PC arm and 2 of 49 (4%; 2/49) participants on the CoE arm have withdrawn from the study before reaching the study end point (*P*>.99). This compares favorably with the anticipated 20% withdrawal rate included in the planning for the trial. One participant on the PC arm and 9 on the CoE are currently on study. As those participants are in follow-up and the randomized trial is still accruing, no data on clinical outcomes is presented.

With 14 of 60 PC and 37 of 50 CoE participants responding, the mean (standard deviation) informed consent knowledge scores, which asked questions about the study purpose, eligibility, expectations regarding study compliance and data reporting, and the ability to withdraw from the study, were 9.8 (88%) and 10.1 (85%), (*P*=.34). In total, 47% of all participants answered more than 91% of the questions correctly. Additionally, 7% of PC participants and 8% of CoE participants answered fewer than 70% of the questions correctly.

### Qualitative Findings

A total of 19 qualitative interviews were conducted with 14 participants recruited from the CoE arm and 5 from the PC arm. Qualitative data illustrated factors driving study enrollment and retention. Participants described a range of inwardly- and outwardly-directed rationales for participating in the study. Outwardly-directed rationales included the stated desire to help others with vasculitis and to further scientific knowledge about the treatment of vasculitis. More inwardly-focused drivers included a sense of a having access to a larger medical network while participating in studies and, specifically for this study, a means of expediting their process of stopping or reducing their prednisone.

Information sources related to study enrollment varied among participants. Some participants described learning about the study through emails or information available on websites (eg, Facebook). Others described finding out about the study through their treating physician. The described information sources were also key factors in patients’ self-assessment of eligibility and appropriateness of testing the study question. Participants described challenges related to study enrollment and some of the specific aspects of the tested study designs. Negotiating risk was a factor in participants’ decision to enroll in the study. Though some participants did not identify risk in study enrollment, others voiced concerns over confidentiality, randomization to an undesired study arm, and concerns over associated risk of flare or sickness. Primary factors driving retention included descriptions of study physicians and study coordinators being viewed as trusted sources of information and having availability to address participants’ concerns about perceived risks. Multimedia Appendix 1 provides illustrative quotes supporting the summative findings above.

## Discussion

### Principal Findings

The Web-based PC approach to recruiting participants into this study was clearly not as successful as the traditional CoE approach. Nonetheless, patients are capable of understanding and correctly appraising whether or not they met the eligibility criteria for the trial and equally astute as to whether their treating physician would agree with the study. Furthermore, there was no difference in the understanding of the trial as reflected in the knowledge surveys of individuals on the PC and CoE arms. In many respects the enrollment yield from the population of potentially eligible participants is the same for the PC or CoE recruitment strategies. The major difference that accounts for the larger number of those enrolled in the PC arm stems from the higher number of eligible subjects.

The ability to get community physicians to facilitate the enrollment process by certifying that either of the TAPIR treatments (prednisone 0 or 5 mg/day) was consistent with the standard of care proved to be problematic. Although eliminating this problem would not have made the accrual rates equal between the arms, it would likely have substantially positively contributed to the accrual numbers. This barrier existed despite the fact that implementation of the PC arm did not require treating community physicians to be involved in the IRB process, nor any more effort other than acknowledging that the treatment choices were medically appropriate, and releasing the medical records. The qualitative findings illustrated that some participants described the physician approval as a both a barrier to enrollment for interested participants and a potential deterrent. Even though not considered part of the study team, PC arm patients ascribed much weight to their treating physician’s opinion about participation and role in or perceived burden of establishing eligibility.

Web-based social media was successful in mobilizing a substantial number of individuals to the study website. Qualitative data supported the intention that these methods would be an efficient way to reach and motivate large numbers of people. The qualitative data suggested that there was interest among participants in the study question as well as an overarching desire to advance knowledge related to the treatment of vasculitis. Others described enrollment as a means of getting off of prednisone where they may not have if not participating in the study. It is possible that increased success of PC recruitment could have been seen with a more broadly applicable or alternative study question. Alternatively, age has been shown to be a moderating factor in the appeal of direct-to-consumer marketing, with older adults relying more on their providers rather than the marketed message [19] which implies that the direct PC appeal of the TAPIR trial might have also have been muted due to the age distribution of this disease.

The prospect of reduced cost of PC recruitment as compared with CoE recruitment, with its need for IRB approvals, continues to make PC recruitment highly appealing. For this particular study question the PC approach was not successful in yielding an accrual rate that could support achievement of the target sample size in a reasonable amount of time and it was substantially less successful than the recruitment achieved by the CoE. Notably, the CoE accrual has also been less than anticipated at the onset of the trial, supporting the conclusion that this trial was a challenge for recruitment in general. The experience of exploring the PC approach in this study yielded several interesting observations and methods that should be evaluated in other clinical settings to get a better sense of the potential of these new methods for clinical trial conduct.

### Conclusions

This study did not suffer from the same issues as the Research on Electronic Monitoring of OAB Treatment Experience (REMOTE) [20] study, which also attempted Web-based recruitment and enrollment in 2011, but it shared a similar fate. The potential benefit of direct-to-patient recruitment remains to be realized and it may be that the characteristics of the best clinical setting and target population are yet to be found.

## Appendix

Multimedia Appendix 1 Qualitative themes and quotations from participant interviews.

## Abbreviations

CoE: center of excellence
GPA: granulomatosis with polyangiitis
PC: patient-centric
RCT: randomized controlled trial
RDCRN: Rare Diseases Clinical Research Network
TAPIR: The Assessment of Prednisone in Remission (TAPIR)
VCRC: Vasculitis Clinical Research Consortium (VCRC)
