# Supplementary material for: Experience With Direct-to-Patient Recruitment for Enrollment Into a Clinical Trial in a Rare Disease: A Web-Based Study
Source: J Med Internet Res. 2017 Feb 28;19(2):e50. doi: 10.2196/jmir.6798 (PMC5350442; doi:10.2196/jmir.6798)
Supplement: Multimedia Appendix 1 [file jmir_v19i2e50_app1.pdf]

Risks
Participants describe risks that a person in the study or considering participation might consider

THEMES		ILLUSTRATIVE QUOTATIONS
Confidentiality	Participants risk sharing important, personal information	"Yeah, I think the risk would be, am I getting the optimal medication that I need as opposed to what the study is prescribing? So that was a concern I had because I wanted to make sure that I was going to getting the medication I needed to, hopefully, stay in remission and be healthy. And I didn't want just to be taking something because I was in the study, right? So that was a concern initially" – CoE<br><br>"That was my biggest concern is getting sick and not being able to do the lower assessment that's required" –PC<br><br>"Certainly, if there's this feeling of security that I'm taking 10 milligrams of Prednisone every day and that's my insurance blanket that, you know, I won't have another flare-up and they're risk-averse, that, you know, going down to five or potentially zero could result in a flare-up" –CoE
Fear or Anxiety	Participants explain that unspecified fear or anxiety might be a risk of participation	
Risk of Staying on Prednisone	Participants describe the potential risk of being randomized to 5mg and staying on prednisone for the duration of the study Participants explain that there was no risk associated with study participation	
Randomization to Undesired Arm	Participants discuss the risk of being randomized to 5mg when they want to be at 0mg or vice versa.	
Risk of Flare or Sickness	Participants describe the risk of flaring while on the 0mg arm	
No Risk	Participants explain that there was no risk associated with study participation	

Eligibility
Participants describe their experience during the eligibility phase of the TAPIR study including descriptions of how eligibility was determined, tapering to the required dosage, and the process they underwent to confirm eligibility.

THEMES		ILLUSTRATIVE QUOTATIONS
Descriptions of the Process to Assess Eligibility	Participants describe the process they underwent to determine the appropriateness of their participation and to ensure that they are in accordance with the eligibility criteria.	"But with weighing the options and going through – my rheumatologist and my ophthalmologist, they worked together closely. They both reviewed the paperwork and they both decided it was okay to do and they've gone through everything." –PC<br><br>"I saw it online, actually, also, because I belong to some Facebook groups that have to do with vasculitis [...] actually, when I saw it, I don't think I was on 5 mg. I think I was on a larger dose. So I don't think it pertained to me at the time. But I think I saw it maybe a month or two before he had told me about it, maybe a few months before" –CoE<br><br>"And then, again, of course, to make sure I had the approval and the participation with my doctor here, too. That's because if he didn't, then I wouldn't have enrolled, because he'd have to work with me on this" –PC<br><br>"To me, he's very knowledgeable and he is the expert. I went to him because my family found him as being the absolute, you know, the doctor's doctor on vasculitis" –CoE<br><br>"I went through a bunch of hoops to get my doctor to say he would participate in it… I gave her the paperwork and stuff, and then I get e-mails from the TAPIR trial thing. They hadn't heard from her, and I'd call her and – or the next time I'd see her. And then eventually she said, well, she had given it to her people in her office to do it, and they would've – they only did them as they came in, and so they were working down the pile to mine" –PC<br><br>""I know it was a two-part consent that you had to agree and that your doctor had to agree. So, maybe people's doctors didn't feel it was a good decision for them" –PC
Physician Addresses Patient's Concerns about Risks	Physician or study staff reassured their patients about the perceived risks of their participation.	
Physicians & Study Coordinators are Trusted Sources	Participants state that the people closely involved in the study are reliable sources that can be used to find out more information about the study.	

Role or Characteristics of Information Sources
Participants describe their sources of information and how those sources affected their decision to participate in the TAPIR study or their experience in the study

THEMES		ILLUSTRATIVE QUOTATIONS
Email or Website Introduction	Participants state that they received an email or looking online was their first introduction to the study and this source may have also provided them with more clarifying information.	"I saw it online, actually, also, because I belong to some Facebook groups that have to do with vasculitis" –PC<br><br>"[My physician] explained that this taper was coming up. He said unfortunately though, you're on five and you have to be at six to ten…" –CoE<br><br>"Basically, I seen it on Facebook, of all places. I belong to the Facebook, Wegener's granuloma vasculitis Facebook chat page, for everybody who has it, or somebody they know who has it, and they've joined. Then we compare things that happened to us versus what we take and how we're treated. And then, of course, seeing that link, that's how I found it, and I decided to try it." –PC
Physician Introduction to study	Participants state that their physician introduced them to the study and provided them with more clarifying information.	

Rationale for Participation in Research
Participants describe their reasons for participating in this research study and general rationale informing their understanding of research

THEMES		ILLUSTRATIVE QUOTATIONS
Comparison to Other Study	Participants compare risks/benefits or purpose between TAPIR and other studies that they have been involved	"Well, primarily, it was the fact that I've been taking prednisone for over a year and I just wanted to be done taking it. And I thought, well, hey, this might actually get me off of it faster. So that was one reason why I was interested in helping" –CoE<br><br>"Maybe they don't want to go through the paperwork with their doctor, and sending the paperwork in, and everything that's involved. Or maybe they don't want to be bothered with the daily log that you go into. It don't bother me" –PC<br><br>"I think you have to have a good discussion with them before you participate in any research. I don't think on-line is the best way to do it because I know a lot of people may be a bit more nervous to enroll for something on-line." –CoE
Further Scientific Knowledge	Participants explain that they enrolled in the present study in order to learn which treatment course is best, including reference about the ways in which best practices are decided and scientific knowledge comes to light.	
Help Others	Participants speak about their participation in the TAPIR study as a means to help others.	
Increased Medical Attention	Participants explain that being part of a study leads to a larger medical support network.	
Participation in Other Study	Participants describe other research studies that they are involved in, including what interested them to join those studies, benefits of study participation, and the study question.	
Stop Prednisone	Participants understood participation in TAPIR as a means to expedite the process of stopping prednisone.	
Potential Challenges	Participants discuss challenges that others might foresee when deciding whether or not to join the study.	
